# Socioeconomic status and quality of life among older adults with hypertension in rural Shandong, China: a mediating effect of social capital

**DOI:** 10.3389/fpubh.2023.1248291

**Published:** 2023-10-17

**Authors:** Yingjie Fu, Shuo Zhang, Xiaolei Guo, Zilong Lu, Xiaojie Sun

**Affiliations:** ^1^Centre for Health Management and Policy Research, School of Public Health, Cheeloo College of Medicine, Shandong University, Jinan, Shandong, China; ^2^National Health Commission (NHC) Key Lab of Health Economics and Policy Research, Shandong University, Jinan, Shandong, China; ^3^Shandong Center for Disease Control and Prevention, Jinan, Shandong, China

**Keywords:** socioeconomic status, quality of life, social capital, older adults with hypertension, rural, China

## Abstract

**Background:**

Improving the quality of life (QoL) of older adults is becoming an important global issue. However, very few studies have been focused on the relationship between socioeconomic status (SES) and QoL in older adults with hypertension. The purpose of this study is to investigate (a) the status of QoL and (b) the mediating effect of social capital in the relationship between SES and QoL, among rural older adults with hypertension in China.

**Methods:**

Using multistage stratified random sampling, a face-to-face questionnaire survey was conducted among rural older adults with hypertension in Shandong province of China from June to July 2021. Three typical measures representing SES were used, namely, annual household income, educational level, and employment status. Individual social capital and QoL were assessed by the Resource Generator-China Scale (RG—China) and a 34-item simplified Patient Report Outcome (PRO)-specific scale for older adults with hypertension, respectively. A total of 950 rural older adults with hypertension were included in the analysis. The mediation model based on bootstrap analyses was employed to explore the relationship between SES and QoL and the mediating role of social capital in the SES-QoL nexus.

**Results:**

The sampled rural older adults with hypertension had an upper-middle level of QoL, and the average score was 132.57 ± 19.40. SES was positively correlated with both QoL and individual social capital; individual social capital was significantly positively correlated with QoL. Controlling for sociodemographic variables, SES was still significantly associated with individual social capital (β = 0.140, *P* < 0.001), and the higher the individual social capital, the better QoL (β = 0.153, *P* < 0.001). Individual social capital played a partially mediating role in the association between SES and QoL (indirect effect = 0.021, *95% CI*: 0.010–0.038), which accounted for 9.38% of the total effect.

**Conclusion:**

This study provides evidence that the effect of SES on QoL was partially mediated by individual social capital among rural older adults with hypertension in China. The government should pay more attention to the rural older hypertensive population with lower SES and strive to reduce the negative impact of poor SES on their QoL, based on effective strategies including improving their individual social capital.

## Introduction

As one of the countries with the fastest population aging, China had 267.36 million people aged older than 60 years in 2020, accounting for 18.9% of the total population (1). It is estimated that the aging population will exceed 500 million, accounting for more than 35.1% by 2050 in China (2). Hypertension is a major public health concern due to its high prevalence and mortality rate, especially in China. According to a report by the World Health Organization (WHO), in 2019, ~60–70% of older adults suffered from hypertension, which is a major risk factor for many cardiovascular, cerebrovascular, and renal diseases (3, 4). The latest data showed that the prevalence rate of hypertension among older adults in China has exceeded 50% (5), and the prevalence rate and growth rate in rural areas were higher than in urban areas (6). However, the rural population was less likely to be aware of, treated for, and controlled for hypertension (6, 7). Therefore, hypertension has become one of the major public health issues in rural China.

With the increasing aging population and the extension of life expectancy, improving the quality of life (QoL) of older adults is becoming an important global issue. As an important indicator of studies on non-communicable chronic diseases (NCDs), QoL is a multidimensional concept encompassing a patient's emotional, physical, and social functional factors (8, 9). Many studies have explored the relationship between QoL and hypertension, which indicated that older adults with hypertension had a lower QoL than the general population (10), and advanced age and comorbidity were negatively associated with QoL (11, 12). In addition, another study in Shaanxi province, China, found that the urban population with hypertension might have better QoL than those from rural areas (13). However, most of the above studies have measured QoL using generic QoL instruments, such as SF-36 and EQ-5D, which did not include important disease-specific domains.

Chinese society is characterized by a stark rural–urban divide due to longstanding disparities in social welfare entitlement, socioeconomic positions, and health profiles between rural and urban residents in later life (14, 15). In fact, in rural China, individuals and families are strongly dependent on mutual social support, when life risks and difficulties occur. Especially for older adults, whose physical condition and working ability are gradually declining and their income is gradually decreasing, traditional rural social network and social capital, embedded in them, are an important guarantee for maintaining a certain standard of living and quality of life. For the rural population with hypertension in China, due to the lack of medical resources, weak health literacy, and health awareness, the extensive interventions and support of socialization forces have a far-reaching impact on their health and QoL. In such a situation, the faster population aging and high prevalence of hypertension in rural areas have posed great challenges to the realization of a healthy China. Therefore, how to improve the QoL of older adults with hypertension in rural areas deserves further attention.

Socioeconomic status (SES), commonly measured by income, education, and occupation, is an overall measure of the economic and social status of an individual or family relative to others (16). SES is also regarded as an important factor in determining an individual's quality of life (17). In the past decades, the relationship between SES and QoL has become an important research issue in the field of healthcare, and similar conclusions on the relationship between SES and QoL were drawn. Low SES has been found to be associated with poor QoL (18, 19). Other studies indicated that chronic disease patients with low SES had low QoL, and there was a differential association between chronic disease conditions and QoL across different SES positions (20, 21). However, there have been very few studies focusing on the relationship between SES and QoL in older adults with hypertension.

Studies on the relationship between social capital and health were mainly based on two different perspectives: “communitarian” and “social network resources”. From the view of communitarian, social capital is defined as the characteristics of social organizations, such as trust, norms, and networks, that can improve social effectiveness by promoting cooperation (22). From the social network resource perspective, social capital refers to the actual or potential resources occupied by the members of a social network or organization, from where the members can get support (23). At present, social capital has been recognized as a social determinant and health influencing factor, as well as an important factor in the prevention and control of chronic diseases (24–26). Studies on the relationship between social capital and the health status of a population with hypertension have disclosed that social capital plays a positive role in the prevention and treatment of hypertension (27, 28). However, very few of them have focused on the relationship between social capital and QoL, especially for those based on the view of social network resources. No study has used a resource generator (RG) scale to measure the individual social capital of older adults with hypertension.

Although previous studies have explored the relationship between SES and QoL, few studies have touched on the role of social capital in this relationship. Some studies have shown that SES not only has a direct impact on health status but also an indirect impact through changes in social capital. However, most of these studies were carried out on the whole older population, adolescents, and urban–rural residents (29–31), while few studies focused on older adults with hypertension. A study in China reported that the effect of SES on QoL is partially mediated through social support among older adults (29). Another study of Chinese Americans also showed that social support mediates the relationship between SES and QoL in breast cancer patients, and improving the level of social support can alleviate the health inequality associated with SES (32). The health ecological model points out that people's health status is determined by individual factors, social factors, and macroenvironmental factors (33). According to the causal distance between the influencing factors and health, the social factors in the health ecological model can be divided into the following aspects: the farthest factor such as macroenvironmental, the second farthest factor such as socioeconomic status, the middle factor such as interpersonal network or social capital, the proximal factor such as behavioral characteristics, and the core factors such as biological factors. Therefore, this study aims to explore the status of QoL and the mediating effect of social capital on the association between SES and QoL among older adults with hypertension in rural China.

## Methods

### Study design and data collection

This study was conducted in Shandong province of China from June to July 2021. Shandong province, located on the eastern coast of China, is the third most economically developed province in China, and it has a population of more than 100 million people, of which 36.95% reside in rural areas (1). In total, 21.22 million people aged older than 60 years lived here in 2020, accounting for 20.9% of the total population (1).

**Figure 1:**
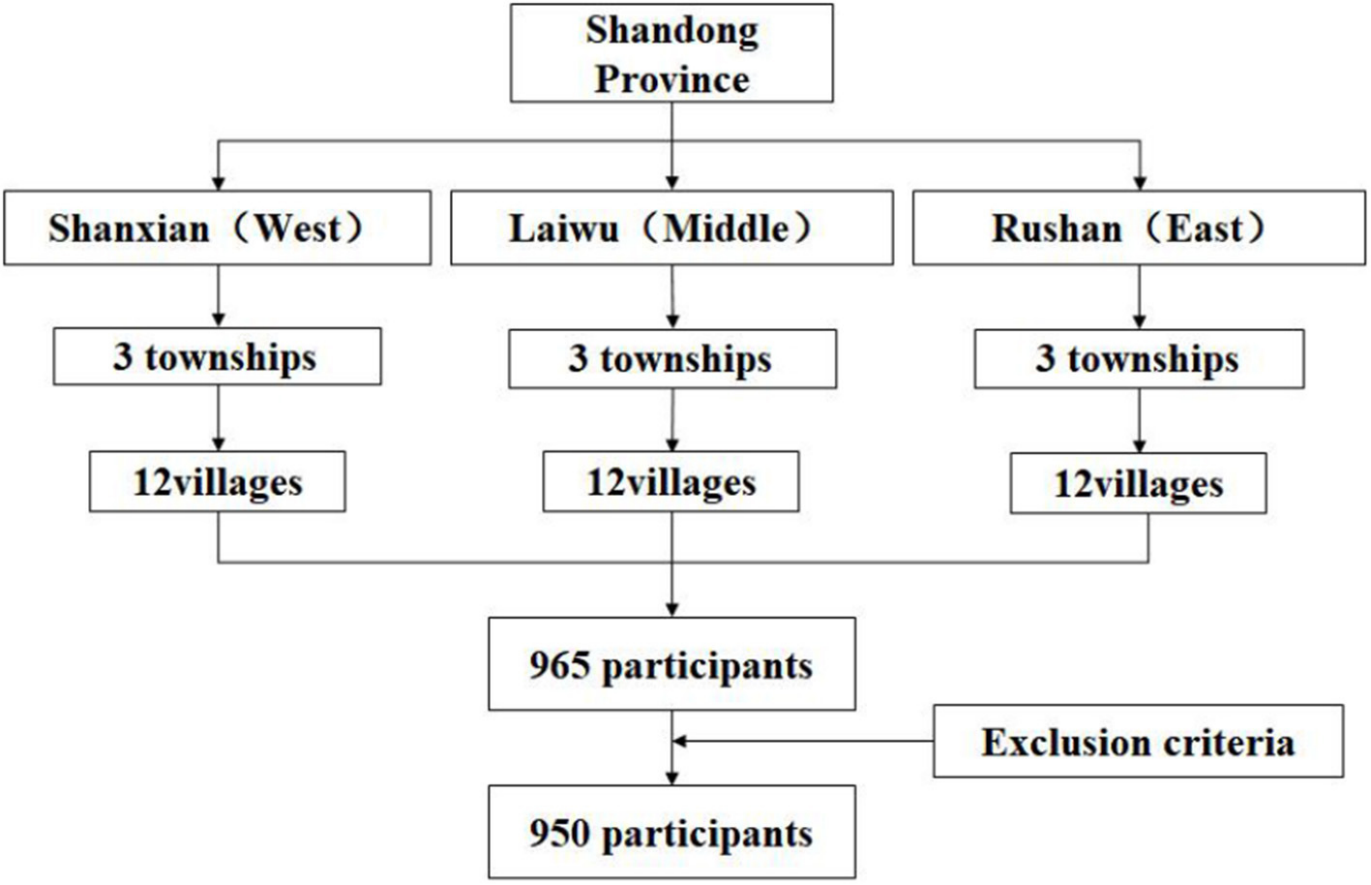
Multistage stratified random sampling framework.

A multistage stratified random sampling framework was used to select participants (Figure 1). According to the socioeconomic levels and geographic location, three provincial counties were selected in Shandong province, namely, Rushan (high economic level, in the east), Laiwu (middle economic level, in the middle), and Shanxian (low economic level, in the west) to obtain a representative sample of older adults with hypertension in rural areas.

The sample size calculation was performed by the following formula: $N=\frac{Z_{\alpha /2}^{2}\times P\left(1-P\right)}{\delta^{2}}$ (N: sample size; ${\text{Z}}_{\alpha /2}^{2}$: confidence interval, 95% confidence level; P: expected prevalence; δ: absolute error, r: relative error, δ = r × P). According to previous studies, the prevalence of hypertension was 53% among Chinese rural older adults (5); so in this study, *P* = 0.53, Z_α/2_= 1.96, δ = 0.06 × *P* = 0.032, α = 0.05. The required sample size was at least 935. Considering the loss and refusal of interviews, the final sample size of the survey was determined to be 960. In each county, older adults with hypertension were randomly selected and identified from the local hypertension registry system. Three townships were randomly selected within each county, and then, four villages were randomly selected within each sample township. Following that, 20–30 older adults with hypertension were randomly selected from each sample village. Finally, 965 older adults participated in the survey, and the number of valid questionnaires was 950 (98.4%).

All investigators received questionnaire survey training a few days before the formal survey. With the assistance of local community workers, skilled and trained teachers and graduate students from the Shandong University of China conducted structured face-to-face questionnaire interviews with the survey participants at the local village clinic or the office of the villagers' committee. The inclusion criteria of the participants were as follows: (1) previously diagnosed with hypertension; (2) at least 60 years old; (3) with an agreement to be investigated; and (4) able to communicate and express normally. The exclusion criteria of the participants were as follows: (1) without an agreement to be investigated; (2) poor mental state caused by a mental disorder or mental illness; and (3) unable to communicate normally due to cognitive impairment or audio–visual impairment.

The Ethics Review Board of the Center for Health Management and Policy Research, School of Public Health, Shandong University approved this study (No. ECCHMPSDU20210404), and it adhered to the tenets of the Declaration of Helsinki. Informed consent was obtained from all participants prior to participating in this study.

### Measures

#### Socioeconomic status

Three different measures of SES were used, namely, household income, educational level, and employment status, which have been extensively studied and validated (34, 35). Household income was collected by asking the participants to report specific income components, such as production income, wage income, transfer income, and others. Then, the respondents were also asked to report their total annual household income for validation of the sum of the components. Household income of participants was ranked from lowest to highest and divided into three groups (high = 2, middle = 1, and low = 0). Considering the actual conditions of older adults in rural China, their educational level was categorized into three groups, namely, middle school and above (high = 2), primary school (middle = 1), and illiteracy (low = 0). Employment status was categorized into three groups, namely, employed = 2, retired = 1, and unemployed = 0. Considering that in rural China, farmers view agricultural production labor as occupational work and the main source of household income, this study classified “engaged in agriculture production at present” as “employed”.

As the three SES indicators in China would not be highly correlated with each other, a single indicator cannot represent the overall SES (36). Therefore, we followed the previous studies to aggregate the above items to get a cumulative SES score (37–40). Scores are assigned to different household income levels, education levels, and employment status (39, 40). The total SES score ranges from 0 to 6, with higher scores indicating a higher socioeconomic status. In this study, the total SES score was classified into four categories, namely, lowest SES (scores ranging from 0 to 1), low SES (scores ranging from 2 to 3), high SES (scores ranging from 4 to 5), and highest SES (score is 6) (39, 40).

#### Quality of life

The 34-item simplified PRO-specific scale for older adults with hypertension, developed by Yang and Wang, was used to measure the health-related QoL of older adults with hypertension in the past week (41). Compared with the generic scale, it can more accurately measure the QoL of older adults with hypertension. The PRO scale is comprised of four dimensions, namely, physiology (11 items), psychology (12 items), society (6 items), and treatment (5 items). The physiology dimension is mainly concerned with physical symptoms and independence and cognitive conditions; the psychology dimension mainly includes paranoia, anxiety, depression, and hostility; the society dimension refers to social support and family role commitment; and the treatment dimension refers to compliance and satisfaction with treatment. It is worth noting that higher scores represent better QoL. The validity and reliability of this PRO scale have been assessed in older adults with hypertension, and it was considered to have good validity and reliability (41). Cronbach's α of this scale in the sample was 0.894.

#### Social capital

The Resource Generator China scale (RG-China), used in this study, has been developed to measure individual social capital for older rural-dwelling adults in China (42). It has been verified that RG-China has good validity and reliability in rural Chinese older adults (43, 44). RG-China has four dimensions, namely, domestic resources (eight items), expert advice (six items), personal skills (six items), and problem-solving resources (five items). Domestic resources refer to resources that can help or improve the quality of daily life; expert advice refers to resources that are helpful for medical treatment, financial management, and agricultural production; personal skills refer to the resources that provide domestic maintenance assistance and enrich entertainment activities; and problem-solving resources refer to resources that help solve personal or family problems and provide moral or material support. RG-China has 25 items, and each item has two levels of response: “yes” or “no”. The scores for the total scale and each sub-scale were calculated, and the higher the score, the more individual social capital. Cronbach's α of this scale in the sample was 0.835.

#### Other variables

Other variables can be classified into five categories in this study, namely, sociodemographic characteristics, health behavior and lifestyle, health service utilization characteristics, disease characteristics of hypertension, and hypertension-related knowledge. Sociodemographic characteristics included gender, age, marital status (married/cohabiting and single including never married, divorced, and widowed), and living arrangement (living alone or with others). Health-related behavior and lifestyle included alcohol drinking, cigarette smoking, physical exercise, BMI (<18.5, 18.5–23.9, 24–27.9, and ≥28), routine physical examination, and medication adherence. Health service utilization characteristics included outpatient and inpatient services received in the past year. Disease characteristics of hypertension included duration of hypertension, multimorbidity, and number of medicines taken. Hypertension-related knowledge of a 12-point scale was used to assess participants' awareness of hypertension treatment and blood pressure control, with scores of ≥7 indicating good awareness and scores of <7 indicating poor awareness (45).

### Statistical analyses

Descriptive summary statistics were estimated for sociodemographic characteristics, such as SES, PRO dimensions, and RG-China dimensions. Means and standard deviations were calculated for continuous variables, and frequencies and percentages were calculated for categorical variables. The non-parametric Kruskal–Wallis test and Wilcoxon rank-sum test were conducted to test the difference in PRO scores among the subgroups, and Spearman correlation coefficients were used to test the correlations between socioeconomic status, social capital, and quality of life variables. All these analyses were performed using Stata 14.0 (Stata Corp, College Station, TX, USA).

**Figure 2:**
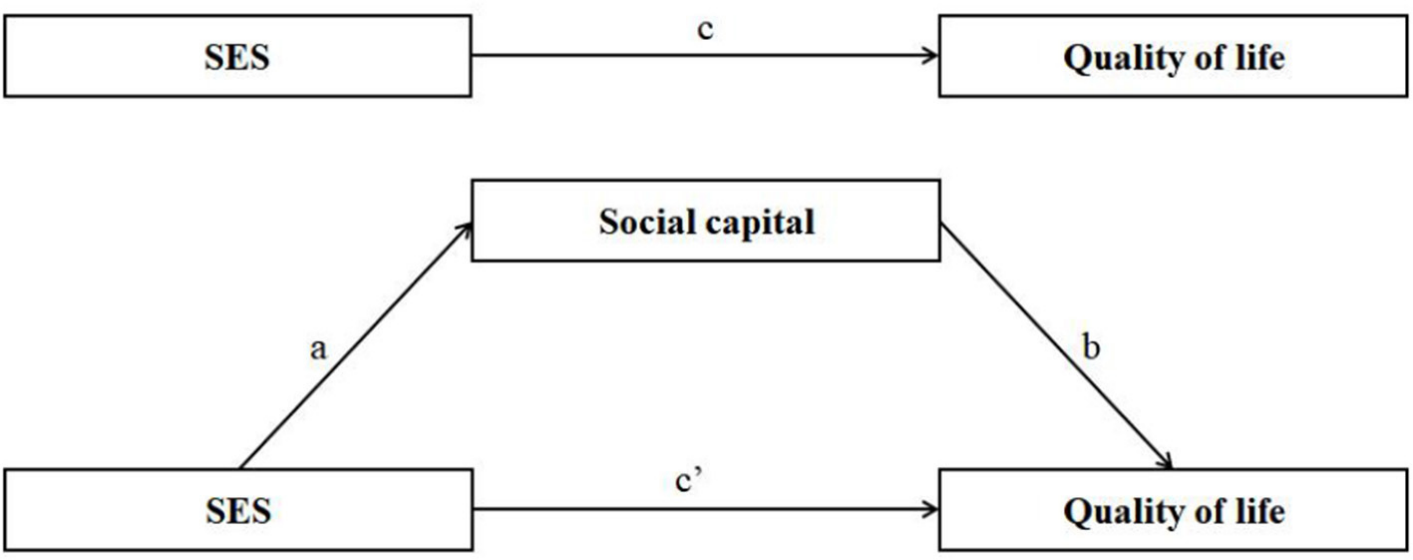
Mediation models. Path a, the coefficient from SES to social capital; path b, the coefficient from social capital to quality of life; path c, the coefficient from SES to quality of life without social capital; path c', the coefficient from SES to quality of life with social capital.

Path analyses with regression function were performed to examine the direct effects of SES (predictor) on quality of life (outcome) and the mediating role of social capital (mediator) in this link. All regression coefficients were tested by the bias-corrected percentile bootstrap method. Specifically, we were interested in (1) the path coefficient from SES to social capital (coefficient a), (2) the path coefficient from social capital to QoL (coefficient b), and (3) the path coefficient from SES to quality of life with a mediator (coefficient c') and without a mediator (coefficient c). The focal parameters are presented in Figure 2. The mediation effect was quantified as a^*^b. We also used a bootstrapping strategy resampled 5,000 times to estimate the bias-corrected and accelerated 95% confidence intervals to test the indirect effects. If the 95% confidence interval did not include 0, it meant that the statistics were significant (46). The mediation model was tested with the PROCESS V.3.3 macro for SPSS. In the current study, model 4 was used to analyze the mediating effect. The model was defined as follows:


$$\begin{array}{l} Y=cX+e_{1}\\ M=aX+e_{2}\\ Y=c^{\prime }X+bM+e_{3} \end{array}$$

where Y is the dependent variable QoL, X is the independent variable SES, and M is the mediator variable social capital; a is the effect of SES on social capital, b is the effect of social capital on QoL, c is the total effect of SES on QoL, and c' is the direct effect of SES on QoL. Specifically, c = c' + a^*^b.

## Results

### Characteristics of the participants

Table 1 displays the characteristics of the participants. Their average age was 70.50 ± 6.11 years, and 61.3% of them were women. The majority of family caregivers had an education level below middle school (73.3%). The annual household income of 20.2% of them was more than 20,000 Chinese Yuan, while 58.0% reported an income level of <10,000 Chinese Yuan. More than half of the participants were employed, and they were mainly engaged in agricultural production labor. The proportions of non-smoking, non-drinking, exercise regularly, BMI > 24, routine health examination every year, and good medication adherence were 72.4, 67.1, 52.7, 72.3, 18.6, and 41.6%, respectively. The vast majority of participants did not utilize outpatient (52.1%) or inpatient (82.5%) services in the past year. In terms of the disease characteristics of hypertension, the proportion of participants also suffering from other diseases was 73.2%; more than half of them (52.6%) had been diagnosed with hypertension more than 10 years; and 67.6% of them took only one type of medicine (67.6%). In addition, approximately half of the participants had good awareness of hypertension treatment and blood pressure control.

**Table 1:** Comparison of quality of life in rural older adults with hypertension by different characteristics.

**Characteristic**	***N* (%)**	**Quality of life score**	**Z/H**	***P*-value**
**Sex**				
Male	368 (38.7)	137.61 ± 19.05	6.82	<0.001
Female	582 (61.3)	129.38 ± 18.96		
**Age**				
60–69	448 (47.2)	132.90 ± 20.07	1.42	0.492
70–79	419 (44.1)	132.65 ± 18.64		
80~	83 (8.7)	130.31 ± 19.60		
**Marital status**				
Single	194 (20.4)	127.04 ± 19.95	−4.5	<0.001
Married/cohabited	756 (79.6)	133.98 ± 19.02		
**Living status**				
Living alone	161 (17.0)	127.63 ± 20.07	−3.6	<0.001
Living with others	789 (83.1)	133.57 ± 19.12		
**Education**				
Illiteracy	353 (37.2)	127.70 ± 19.03	47.6	<0.001
Primary school	343 (36.1)	133.87 ± 18.37		
Middle school and above	254 (26.7)	137.57 ± 19.80		
**Annual household income**				
<10,000 Chinese Yuan	551 (58.0)	128.89 ± 19.60	56.04	<0.001
10,000–20,000 Chinese Yuan	207 (21.8)	135.57 ± 17.92		
≥20,000 Chinese Yuan	192 (20.2)	139.88 ± 17.75		
**Employment status**				
Employed	515 (54.2)	135.35 ± 18.33	57.59	<0.001
Retired	61 (6.4)	142.69 ± 17.15		
Unemployed	374 (39.4)	127.08 ± 19.75		
**Cigarette smoking**				
Non-smoking	688 (72.4)	130.38 ± 19.11	36.06	0.001
Former smoking	146 (15.4)	138.65 ± 19.03		
Current smoking	116 (12.2)	137.86 ± 19.12		
**Alcohol drinking**				
Non-drinking	637 (67.1)	130.48 ± 19.25	38.14	<0.001
Former drinking	122 (12.8)	131.46 ± 21.26		
Current drinking	191 (20.1)	140.23 ± 16.68		
**Physical exercise**				
Never	349 (36.7)	130.12 ± 20.32	12.6	0.002
Occasional	100 (10.5)	130.53 ± 18.02		
Regular	501 (52.7)	134.67 ± 18.80		
**BMI, kg/m** ^2^				
<18.5	16 (1.7)	127.56 ± 19.18	4.87	0.181
18.5–23.9	248 (26.1)	133.93 ± 19.66		
24–27.9	411 (43.3)	132.81 ± 19.39		
≥28	275 (29.0)	131.27 ± 19.18		
**Routine physical examination**				
Yes	177 (18.6)	135.90 ± 19.90	2.93	0.003
No	773 (81.4)	131.80 ± 19.22		
**Medication adherence**				
No medicine	22 (2.3)	134.95 ± 17.09	1.26	0.533
Poor adherence	533 (56.1)	132.06 ± 19.14		
Good adherence	395 (41.6)	133.12 ± 19.89		
**Outpatient treatment status in the past year**				
Yes	455 (47.9)	130.04 ± 20.13	−3.69	<0.001
No	495 (52.1)	134.89 ± 18.43		
**Inpatient treatment status in the past year**				
Yes	166 (17.5)	127.37 ± 20.10	−3.68	<0.001
No	784 (82.5)	133.67 ± 19.08		
**Duration of hypertension**				
≤ 5 years	205 (21.6)	136.82 ± 17.59	29.46	<0.001
6–10 years	245 (25.8)	135.46 ± 19.44		
≥11 years	500 (52.6)	129.40 ± 19.57		
**Multimorbidity**				
Yes	695 (73.2)	129.3 ± 19.44	−8.9	<0.001
No	255 (26.8)	141.48 ± 16.28		
**Number of medicines taken**				
1	642 (67.6)	132.85 ± 19.00	3.85	0.146
2	221 (23.3)	133.39 ± 19.39		
≥3	87 (9.2)	128.39 ± 21.93		
**Hypertension-related knowledge**				
<7	463 (48.7)	131.11 ± 18.91	−2.67	0.008
≥7	487 (51.3)	133.95 ± 19.78		

Z, Z-value of Wilcoxon rank-sum text; H, H-value of non-parametric Kruskal-Wallis test.

### Quality of life in rural older adults with hypertension with different characteristics

The QoL scores of most characteristic subgroups were distributed between 127 and 142. Statistical differences were revealed in the other variables except age, BMI, medication adherence, and number of medications taken. The participants had higher QoL scores, who belonged to the following characteristic subgroups: males, married/cohabited, living with others, having an education level of middle school and above, having a high annual household income, retired, former smoking, current drinking, physical exercise regularly, routine health examination every year, not utilizing outpatient or inpatient services in the past year, with hypertension <5 years, with no multimorbidity, and having good knowledge about hypertension.

### Description analyses of SES, quality of life, and social capital

The mean score of the SES was 2.67 ± 1.70, and the proportions of lowest SES, low SES, high SES, and highest SES were 26.1, 41.5, 27.4, and 5.1%, respectively. The mean scores of the total social capital and four sub-dimensions were 18.66 ± 4.14, 6.98 ± 1.79 (domestic resource), 3.92 ± 1.52 (expert advice), 3.25 ± 1.46 (personal skills), and 4.51 ± 1.04 (problem-solving resources), respectively. The mean scores of the QoL and four sub-dimensions were 132.57 ± 19.40, 41.99 ± 8.32 (physiology), 49.86 ± 9.84 (psychology), 22.83 ± 4.49 (society), and 17.89 ± 4.98 (treatment), respectively.

### Correlation analyses of SES, quality of life, and social capital

Table 2 provides the results of correlation analyses for SES, social capital, and QoL, respectively. SES was positively correlated with QoL (β = 0.330, *P* < 0.001) and social capital (β = 0.181, *P* < 0.001), respectively. Social capital was significantly positively correlated with QoL (β = 0.196, *P* < 0.001).

**Table 2:** Spearman correlation coefficients between key study variables.

**Variables**	**SES**	**Social capital**	**QoL**
SES	1		
Social capital	0.181^***^	1	
QoL	0.330^***^	0.196^***^	1

^***^P <0.001.

### Analyses of the mediating effects

As shown in Table 3, three models were constructed to test the mediating role of social capital in the relationship between SES and QoL. Model 1 took SES as an independent variable, and QoL as the dependent variable. After controlling for other variables, SES was significantly associated with QoL (β = 0.227, *P* < 0.001). In model 2, SES was still taken as an independent variable, while social capital was used as a dependent variable. The results of model 2 showed that SES had a significant positive effect on social capital (β = 0.140, *P* < 0.001). When including social capital in model 3, the association between SES and QoL was still statistically significant, while the β coefficient decreased by 0.021 (β = 0.206, *P* < 0.001), compared with that in model 2; social capital was also significantly associated with QoL (β = 0.153, *P* < 0.001).

**Table 3:** Testing the mediation effect of social capital between SES and QoL among the participants in Shandong, China.

**Predictors**	**Model 1 (QoL)**		**Model 2 (Social capital)**		**Model 3 (QoL)**	
	β	***t***	β	***t***	β	***t***
SES	0.227	6.964^***^	0.140	4.145^***^	0.206	6.252^***^
Social capital					0.153	4.831^***^
Gender	−0.159	−1.660	0.388	3.470^***^	−0.219	−2.282^*^
Marital status	0.095	0.772	−0.077	−0.531	0.107	0.864
Living status	−0.006	−0.045	0.097	0.595	−0.020	−0.160
Cigarette smoking	0.002	0.040	0.260	3.750^***^	−0.037	−0.649
Alcohol drinking	0.058	1.226	0.115	2.244^*^	0.041	0.876
Physical exercise	0.096	2.927^*^	0.188	5.354^***^	0.067	2.076^*^
Routine physical examination	−0.231	−3.007^*^	0.334	3.600^***^	−0.282	−3.681^***^
Outpatient treatment status	0.180	3.008^*^	−0.036	−0.565	0.186	3.125^**^
Inpatient treatment status	0.162	1.899	0.217	2.260^*^	0.129	1.519
Duration of hypertension	−0.117	−3.286^**^	0.021	0.537	−0.120	−3.387^***^
Multimorbidity	0.507	7.816^***^	0.034	0.463	0.502	7.882^***^

Variables with significant effects on quality of life in univariate analysis were adjusted, including gender, marital status, living status, cigarette smoking, alcohol drinking, physical exercise, routine physical examination, outpatient treatment status in the past year, inpatient treatment status in the past year, duration of hypertension, and multimorbidity.

^***^P <0.001.

^**^P <0.01.

^*^P <0.05.

Therefore, we supposed that social capital may mediate the relationship between SES and QoL. The total effect, direct effect, and indirect effect of SES on QoL, with social capital as a mediator, are presented in Table 4. After controlling for potential confounders, the bootstrapping method was used to further verify the mediation effect, and the direct effect, indirect effect, and total effect were all statistically significant. Social capital played a partial mediating effect in the association between SES and QoL (indirect effect = 0.021, *95% CI*: 0.010–0.038), and the mediating effect of social capital accounted for 9.38% of the total effect.

**Table 4:** Total effect, direct effect, and indirect effect of SES on QoL with social capital as mediator.

	**Effect size**	**Boot SE**	**Boot 95%CI (low, high)**	**Relative effect value**
Total effect	0.227	0.033	(0.163, 0.291)	
Direct effect	0.206	0.033	(0.141, 0.271)	90.62%
Indirect effect	0.021	0.007	(0.010, 0.038)	9.38%

Boot SE, bootstrap standard error.

## Discussion

To the best of our knowledge, this is the first study to explore the association of SES with QoL in rural older adults with hypertension in China, as well as the mediating effect of social capital. Our study demonstrated that SES was associated with QoL, and the effect of SES on QoL was partially mediated by social capital among rural older adults with hypertension.

The present study quantified the QoL of rural older adults with hypertension by using the PRO-specific scale for older adults with hypertension. The sampled rural older adults with hypertension had an upper-middle level of QoL, which was not completely consistent with previous studies using hypertension patient-specific QoL scales. A study used the QLICD-HY scale (quality of life instrument for a population with hypertension) to measure the QoL of the hypertensive population in Shaoyang city of Hunan province, which had a similar finding to this study (47). However, another study using the same scale found a lower level of QoL in the hypertensive population in Shanxi province (48). Focusing on older adults with hypertension, a study using the QLICD-HY scale reported that older adults with hypertension in Nanjing city had a higher QoL (49). In addition, most of the previous studies measured the QoL of hypertension using generic instruments and indicated that the rural hypertension population might have lower QoL than those in urban areas (13). The QoL in this study was inconsistent with other studies, which can be explained in the following three aspects. First, participants in the current study were older adults aged 60 years and older, while many previous studies included all adults older than 18 years. Second, there were some differences in the applicable population among different specific scales. Our study used the specific scale for older adults with hypertension, while the QLICD-HY scale was targeted at all age groups of the hypertension population. Finally, regional differences and urban–rural differences also affected the QoL of the population with hypertension.

This study revealed that SES was positively associated with QoL among rural older adults with hypertension. In other words, the Chinese rural older hypertensive population with a high level of SES had better QoL. This finding was consistent with previous research in Chinese older adults (50). Focusing on the population with hypertension, a study on the relationship between SES and mental health in the hypertensive population in Shandong province, China, reported that SES was positively correlated with mental health, which measured the SES by education background, occupation, and household income (39). Another study also showed that the risk of hypertension was different among people with different SES levels, the detection rate of hypertension was higher among people with lower education level, and the prevalence of hypertension in low-income people was higher than that in high-income people (51).

Our results indicated that there was a significant positive association between SES and social capital among rural older adults with hypertension. Similar findings have been disclosed by previous studies among adolescents, older adults, or rural–urban residents. A longitudinal study focusing on the impact of residents' SES on social capital, based on the data from China's General Social Survey, found that people with lower SES had worse social capital (52). Studies in other countries also indicated that people with higher SES had large-scale, high-quality, and more robust social relationship networks. Two possible reasons may be explained as follows: on one hand, SES determines the environment in which people live and work and the accessibility of health products and services (53). On the other hand, SES also affects people's psychological state and perception of the world around them (54).

Previous studies have shown that there was a positive relationship between social capital and QoL among different population groups, including older adults, family caregivers, and patients with stroke, HIV/AIDS, and others (50, 55–60). Similarly, this study disclosed that social capital was linked to the QoL among rural older adults with hypertension. This finding is in line with a previous study from a cross-national analysis, which suggested that hypertensive population with a higher individual social capital had better hypertension management, thereby improving their QoL (28). Another longitudinal follow-up survey in rural China reported that baseline individual social capital positively affects the QoL in older adults with hypertension (44). However, the above studies were based on the communitarian view, without taking into account actual or potential resources within social networks. Therefore, through measuring social capital with RG-China, our study enriches the existing research on the relationship between social capital and QoL in the hypertensive population.

Furthermore, this study revealed that social capital played a significant mediating role between SES and QoL among rural older adults with hypertension, which is vital for improving QoL. The rural older adults with hypertension with higher SES were more likely to have more social network resources, which were associated with better QoL. With the improvement in SES, people's experiences of social activities will become richer, which is conducive to expanding their social network and enhancing their social capital. Higher social capital represents a higher frequency of social participation, a higher level of social trust, and a higher quality of material and emotional support (61, 62). On one hand, adequate social participation in daily life not only promotes physical and mental activities but also enhances daily activities, such as ability and cognition. On the other hand, social capital can also alleviate anxiety and depression. Therefore, social capital is a beneficial factor to improve QoL in both physical and psychological aspects. These findings suggest that the effects of SES on QoL were partially mediated by social capital. Based on the above results and the high prevalence of hypertension among older adults in rural China, it is imperative to take multifaceted management strategies to prevent and control hypertension among older adults. The rate of missed diagnosis of hypertension in China is as high as 57%, and only 9% are properly treated (63). Studies have shown that the low socioeconomic status groups are more likely to delay the diagnosis and treatment of hypertension (64). Therefore, the results of this study also have a certain reference value for rural older adults with undiagnosed hypertension. We suggest that primary healthcare should strengthen the screening for chronic non-communicable diseases such as hypertension among older adults, especially in those with low SES. Health providers can regularly carry out health education to provide disease prevention, treatment, and self-management knowledge for rural older adults. In addition, it is necessary for the rural communities to build some public facilities and activities and encourage the older adults to participate in social activities, so as to improve their social capital as possible.

Although this study extends the existing literature on QoL among rural older adults with hypertension, there are still several limitations to acknowledge. First, given the cross-sectional survey, the present analyses cannot identify the causal relationships between SES, social capital, and QoL. Further research will be conducted to explore the causality with longitudinal survey data. Second, our data were collected using a structured questionnaire based on self-reported information of the participants, which might be subjected to some recall bias. In addition, the measurement of SES in our study only included household income, educational level, and employment status and did not include other indicators that can measure SES. In future studies, we will consider including other indicators that can measure SES. Third, this study was conducted in rural areas of Shandong province, China, and caution should be taken when extending these results to other rural regions. However, owing to the many similarities of rural China regions, the results of this study still have relatively good reference value.

## Conclusion

This study demonstrated that SES was positively associated with QoL, and the effect of SES on QoL was partially mediated by social capital among older adults with hypertension in rural China. The government and policymakers should pay more attention to those with lower SES and strive to reduce the negative impact of poor SES on their QoL, based on effective strategies including improving their social capital.

## Data availability statement

The original contributions presented in the study are included in the article/supplementary material, further inquiries can be directed to the corresponding author.

## Ethics statement

The Ethics Review Board of the Center for Health Management and Policy Research, School of Public Health, Shandong University approved this study (No. ECCHMPSDU20210404), and it adhered to the tenets of the Declaration of Helsinki. Informed consent was obtained from all participants prior to participate in this study.

## Author contributions

XS: conceptualization, critical revision, funding acquisition, supervision, writing—original draft preparation, and writing—reviewing and editing. YF: data curation, formal analysis, software, critical revision, writing—original draft preparation, and writing—reviewing and editing. SZ: data curation, formal analysis, and supervision. XG: data curation and supervision. ZL: data curation and supervision. All authors contributed to the article and approved the submitted version.
